# Performance Study of Stabilized Recycled Aggregate Base Material with Two-Gray Components

**DOI:** 10.3390/ma17205038

**Published:** 2024-10-15

**Authors:** Kai Wang, Xianhu Hu, Yingjie Yuan, Feng Lian, Mingchen Zhong, Kun Meng

**Affiliations:** 1 College of Transportation, Shandong University of Science and Technology, Qingdao 266590, China202001130327@sdust.edu.cn (M.Z.); 2 Qingdao Greensail Recycled Building Materials Co., Ltd., Qingdao 266043, China; 202001130308@sdust.edu.cn; 3 Shandong Academy of Building Sciences Co., Ltd., Qingdao 266000, China201901130517@sdust.edu.cn (F.L.)

**Keywords:** construction waste, lime fly ash, base material, test section, pavement performance

## Abstract

This article studies the practical road performance of recycled materials from construction waste, relying on the paving test section of the supporting project for the Qingdao Cross-Sea Bridge. The research focuses on the construction technology and road performance of using recycled construction waste materials in urban road sub-base construction. Through indoor tests such as sieving and unconfined compressive strength tests, relevant technical indicators were obtained and analyzed. Additionally, periodic core sampling, compaction tests, and rebound deflection tests were conducted on-site according to relevant standards to thoroughly investigate the specific effects of using construction waste in practice and to analyze and evaluate the actual feasibility of the materials for road use. The results indicate that the particle gradation of the construction mix in the test section aligns well with the target gradation, and the dosage of the mixing agent meets the design requirements. The 7-day unconfined compressive strength already satisfied the technical requirements for heavy and extremely heavy traffic on highways as specified in the “Technical Specifications for Construction of Highway Pavement Subbase” (JTG/T F20-2015), with the 14-day strength generally reaching 7 MPa. Core sampling revealed good aggregate gradation, smooth and straight profiles, and the thickness and strength of all parts meet the specifications. The compaction levels met the testing requirements, the surface deflection values showed a decreasing trend, and the deformation resistance was good, consistent with the general development patterns of semi-rigid sub-bases.

## 1. Introduction

In the past 30 years, driven by the rapid development of urbanization in our country, the scale of construction has continually expanded, resulting in a continuous generation of massive amounts of construction waste [1]. This has made environmental issues particularly prominent, prompting research into the secondary utilization of waste across various innovative fields [2,3]. At the same time, the scale of transportation infrastructure construction is further expanding. The gravel and other materials used in highway engineering construction have problems such as soaring prices and insufficient stock. At the same time, excessive exploitation will also have a bad impact on the ecological environment, which is not conducive to the sustainable development of transportation power. Therefore, it is not only an important research direction at present [4,5,6,7,8] but is also in line with the current actual situation to use the inorganic binder-stabilized crushed stone base material prepared using the recycled aggregate of construction waste for the preparation of highway engineering materials, which is of great significance to improve the ecological environment and alleviate the shortage of natural resources.

Shah et al. [9] studied the physical properties of recycled aggregate from construction waste through experiments. The research found that the load-bearing capacity of the aggregate meets the technical requirements and possesses excellent particle size distribution and durability compared to natural materials. Ming [10] investigated the application of recycled concrete aggregate derived from construction waste in road sub-bases, discovering that the water absorption rate of the recycled aggregate is three times that of the natural aggregate. Although there are differences in density, crushing value, and Los Angeles abrasion value compared to the natural aggregate, they all meet the standard requirements. Giwangkara et al. [11] examined the feasibility of using recycled aggregate to prepare materials for road sub-bases, conducting tests on density, water absorption, aggregate crushing value (ACV), compaction, and permeability (CBR). The results indicated significant differences compared to the natural aggregate, leading to the suggestion of using additives to improve the recycled aggregate. Kang et al. [12] explored the feasibility of using mixtures made from recycled aggregate for road sub-bases, finding that its drainage characteristics outperformed those of a mixture made with 100% natural aggregate and exhibited greater cohesion. Limbachiya et al. [13] studied the effect of recycled coarse aggregate content on the strength of mixtures, revealing that when the content of recycled coarse aggregate exceeds 30%, the strength gradually decreases with increasing content. They proposed adjusting the cement ratio to enhance the recycled coarse aggregate content and demonstrated that the strength and durability of the resulting mixture were comparable to that of natural aggregate mixtures.

Yang et al. [14] and Esfahani [15] studied the mechanical and road performance of recycled aggregate mixtures, analyzing the impact of factors such as the proportion of recycled aggregates on the strength of road sub-base materials. Luo et al. [16] estimated the elastic modulus of recycled aggregate mixtures based on material performance characteristics and discrete element simulation tests. The results indicated that the simulation tests were consistent with laboratory tests, showing that the materials had high water absorption and low durability, with elastic properties increasing with confining pressure. Meng et al. [17] investigated the feasibility of using 100% construction waste recycled aggregates in cement-stabilized materials, while Kong et al. [18] prepared recycled crushed stone aggregates using two types of fly ash, finding that they could serve as semi-rigid aggregates, although their maximum dry density was lower and the optimal moisture content was higher. Kolay et al. [19] examined the effect of recycled aggregate content on the strength of concrete mixtures and compared it with mixtures made from natural aggregates. The results revealed that the proportion of recycled aggregates significantly affected the strength and other properties, with mixtures made from recycled aggregates showing lower CBR values. Janile [20] studied the feasibility of using two different types of recycled concrete to prepare road sub-base materials, finding that the mixtures exhibited good properties in terms of strength, elastic modulus, permanent deformation, and durability. They also discussed and analyzed the effects of curing time, liquid-to-solid ratio, and particle size on the metal leaching of recycled materials.

Currently, a large amount of research is focused on the feasibility of preparing and verifying stabilized crushed stone base materials using recycled aggregates, with a lack of performance studies on experimental sections. Additionally, there has yet to be widespread application in urban roads, and the construction processes and quality control standards are not yet well established. Tavira et al. [21] studied the mechanical properties of laboratory and field non-bound materials made from mixed recycled aggregates derived from construction waste, providing preliminary verification of feasibility for practical road use. Therefore, this paper aims to investigate the road performance of recycled materials from construction waste, utilizing the pavement test section for the Qingdao Cross-Sea Bridge supporting project to conduct research on the construction technology and road performance of recycled materials for urban road base layers. The base and sub-base materials in the test section were prepared by replacing 100% of natural aggregates with recycled aggregates from construction waste in the production of cement-stabilized crushed stone base materials. First, through indoor tests including particle size analysis, gray dosage tests, unconfined compressive strength tests (1 d, 3 d, 7 d, 14 d, 28 d), and freeze–thaw resistance tests on the materials used in the test section, relevant technical indicators were obtained and analyzed. Then, after the construction of the test section, periodic core sampling, compaction measurements, rebound deflection, and other field tests were conducted in accordance with relevant specifications to evaluate the actual road performance of the materials. Lastly, by utilizing initial indoor tests and subsequent pavement inspections, a thorough investigation into the practical effects of using construction waste was carried out, providing a comprehensive evaluation of the materials’ actual feasibility for road use.

## 2. Materials and Method

### 2.1. Material Type and Design

**Figure 1:**
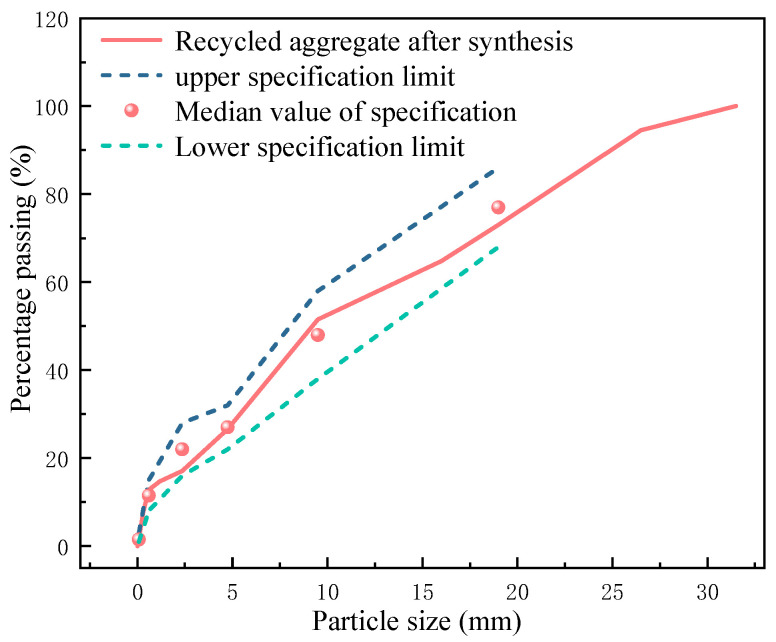
Grading curve.

Based on extensive previous research, inorganic mixtures stabilized with cement and lime can meet the strength requirements specified in regulations. From both environmental and economic perspectives, the base course and sub-base materials for this test section use 100% recycled aggregates from construction waste to replace natural aggregates. The composition of the materials includes lime, fly ash, and recycled aggregates in proportions of 8%, 17%, and 75%, respectively. The recycled aggregates come from waste concrete sourced in Qingdao and are processed by Qingdao Lufan Recycled Building Materials Co., Ltd. (Qingdao, China). They mainly consist of discarded concrete, bricks, and a small number of tiles, classified into three specifications based on particle size: recycled fine aggregates of 0~5 mm and recycled coarse aggregates of 5~10 mm and 10~25 mm. According to earlier studies, the synthesis proportions of the recycled aggregates are 24%, 26%, and 50% for the 0~5 mm, 5~10 mm, and 10~25 mm categories, respectively, with the grading curve shown in Figure 1. The lime used is grade III slaked lime produced by Shandong Yuxin Nano Technology Co., Ltd. (Weifang, China), with an effective calcium and magnesium content totaling 57.33%. The fly ash used is grade II fly ash produced by Huadian Laizhou Power Generation Co., Ltd. (Yantai, China), with a loss on ignition of 6.7%, and a total content of SiO_2_, Al_2_O_3_, and Fe_2_O_3_ of 83.11%. The physical and chemical properties of the raw materials used in the test section meet the regulatory requirements as confirmed by prior testing.

### 2.2. Research Protocol

The test section relies on the supporting project of the Qingdao Cross-Sea Bridge, with a total length of 230 m and a width of 19.6 m, featuring six lanes in both directions. The test section is set within the ZK0 + 220 to ZK0 + 320 segment of the project road. First, inorganic materials are produced using mixing equipment at the inorganic material mixing plant, transported to the site, and laid and compacted using the same construction methods as those for conventional inorganic material road bases. Then, during construction, relevant materials are sampled for lab tests according to specifications, including grading tests, compaction tests, optimal moisture content tests, and unconfined compressive strength tests (1 d, 3 d, 7 d, 14 d, 28 d, where d means days) to obtain and analyze the relevant technical indicators of the materials. Finally, on-site inspections are conducted in accordance with relevant specifications, including periodic core sampling (for thickness and strength), compaction testing (using the sand replacement method), and rebound deflection measurements. The appearance and integrity of the samples are observed, and in conjunction with the results of the lab tests, the material performance of the test section and the feasibility of using a stabilized crushed stone base with secondary gray materials are analyzed and evaluated.

**Figure 2:**
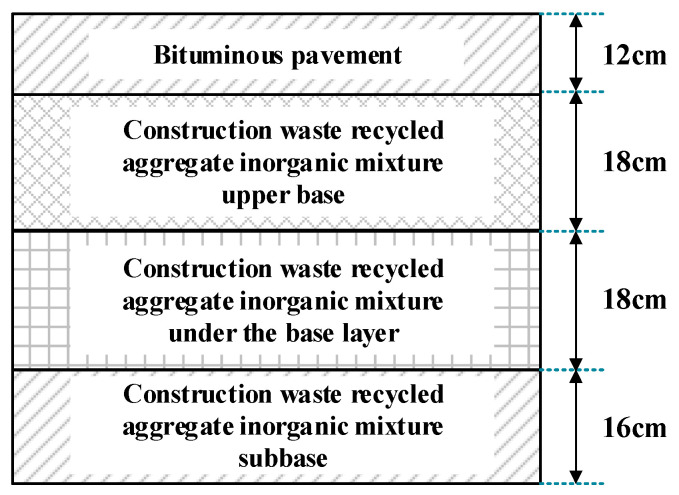
Schematic diagram of test section structure.

Based on the road class, traffic volume design, and the reuse of waste materials, the structure of the sub-base and base course for the test road section is proposed, as shown in Figure 2. The stabilized inorganic mixture is laid in three layers: an upper base layer of 18 cm, a lower base layer of 18 cm, and a bottom base layer of 16 cm. Using high-strength recycled coarse aggregates in the bottom base layer can improve the interlocking capability of the mixture and enhance the load-bearing capacity.

### 2.3. Test Method and Construction Technology

Before mixing the materials for the test section, an appropriate amount of prepared recycled aggregate should be taken and a screening test should be conducted following the test methods specified in the “Test Methods for Aggregates in Highway Engineering” (JTG 3432-2024) [22]. After mixing, an appropriate amount of inorganic mixture should be taken, and tests for compaction, ash dosage, unconfined compressive strength (1d, 3d, 7d, 14d, 28d), and freeze–thaw resistance should be conducted according to the test methods specified in the “Test Methods for Inorganic Binders Stabilized Materials in Highway Engineering” (JTG 3441-2024) [23]. Finally, after laying and compacting, curing should be carried out by watering, and periodic core sampling, in situ compaction detection, and rebound deflection testing should be performed in accordance with the test methods specified in the “Field Testing Procedures for Roadbed and Pavement in Highway Engineering” (JTG 3450-2019) [24].

**Figure 3:**
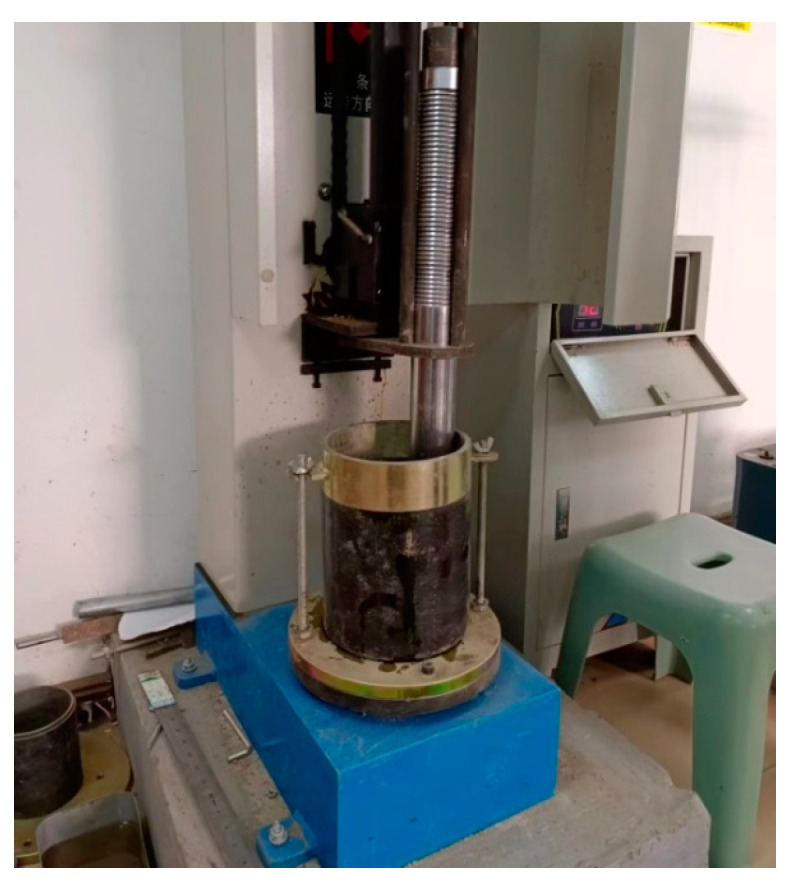
Compaction test.

**Figure 4:**
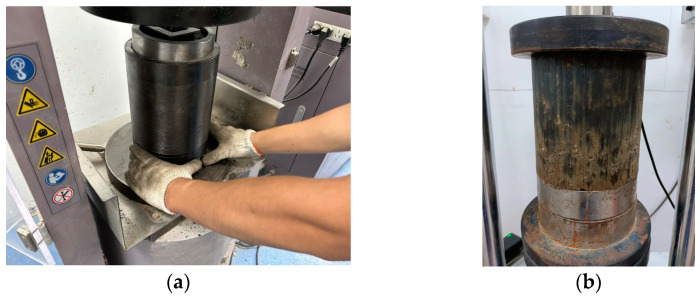
Unconfined compression strength test: (**a**) hydrostatic forming; (**b**) pressure application.

**Figure 5:**
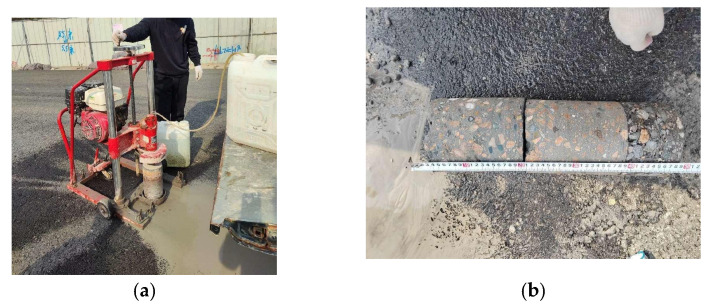
Coring inspection: (**a**) pavement coring; (**b**) core thickness detection.

Some of the tests and inspections are illustrated in Figure 3, Figure 4 and Figure 5. In the compaction test, a heavy compaction method is used for the inorganic mixture applied in the test section, setting up three specimens and taking the average of the results. The hammer weighs 4.5 kg, the drop height is 45 cm, and the number of blows per layer is 98, with three layers compacted. The volume of the test cylinder is 2177 cm^3^. In the ash dosage test, the EDTA titration method is used to examine the mixture, carrying out two tests per layer for a total of six tests. The unconfined compressive strength indicates the capacity of the base material specimens to withstand axial pressure in the absence of lateral pressure and is an important parameter for assessing the mechanical properties of semi-rigid base materials. The mixture used in the test section is formed into specimens for unconfined compressive strength testing at 1 d, 3 d, 7 d, 14 d, and 28 d, with 13 Φ150 mm × 150 mm specimens prepared for each age per layer, totaling 195 specimens, and the average for each age in each layer is taken. In the freeze–thaw resistance test, nine specimens of the mixture used in the test section are prepared for five freeze–thaw cycles to assess the material’s freeze resistance. To evaluate the curing status of the test section, the first core sampling is conducted on the 7th day after the material is laid, and the second core sampling is conducted on the 30th day post-laying. In the compaction detection, two tests are conducted on the test section, with five measurements taken per structural layer each time. For the rebound deflection testing, measurements are taken for the subgrade before laying and on the 9th day after laying the base layer.

The construction process of the test section is fundamentally similar to that of ordinary inorganic materials, which mainly includes mixing, transportation, paving, compaction, and curing. Given the significant variability in the performance of construction waste, strict performance testing is conducted on raw materials such as recycled aggregates from waste concrete, lime, and fly ash. Indoor tests are carried out to examine and validate the compaction properties, unconfined compressive strength, and other characteristics of the inorganic blend. The inorganic blend must meet the requirements of no obvious segregation of coarse and fine aggregates and consistent coloration of the mix. During transportation, measures are implemented to prevent spillage and water loss of the produced blend. The paving and compaction process for the base materials is identical to that of ordinary inorganic materials, with a paving machine used for spreading at a speed controlled between 1.0 and 1.5 m/min. The on-site distribution is relatively uniform, and the compaction effect is good, indicating that the recycled inorganic blend from construction waste has favorable compactness, stability, and uniformity. The paving of the lime–fly ash-stabilized recycled aggregates in the test section was carried out in October 2023. After the laying was completed, traffic was closed, and watering for curing was conducted for 7 days.

## 3. Results and Discussion

### 3.1. Comparative Verification

**Figure 6:**
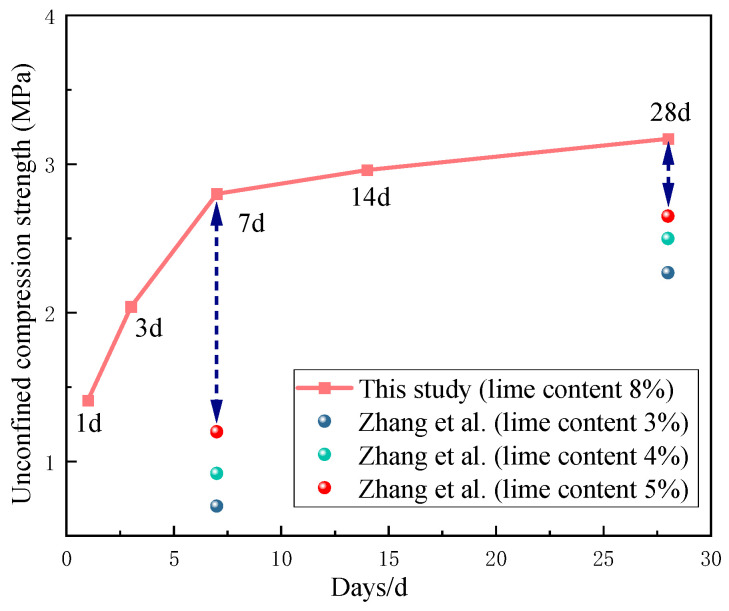
Comparison between proposed solutions and solutions obtained by Zhang et al [25]: Unconfined compressive strength.

Figure 6 compares the unconfined compressive strength of the proposed mix method with previous research results (Zhang et al. [25]), where the type of recycled aggregate from construction waste is all discarded concrete. In this study, the contents of lime and fly ash used are 8% and 17%, respectively, while the compared study (Zhang et al.) used a fly ash content of 12% and lime contents of 3%, 4%, and 5%. As shown in Figure 6, in the study by Zhang et al., the 7-day unconfined compressive strength can reach 0.72–1.18 MPa. The unconfined compressive strength increases with the increase in lime content and curing period, and the effect of lime content is significant. When the lime content is 4%, it can be used for pavement structures requiring a strength of 0.8 MPa. When the lime content is 5%, it can be used for pavement structures with higher strength requirements. The study by Zhang et al. also conducted durability tests; when the lime content was 4%, a freeze-thaw cycle test was performed at 28 days of curing, resulting in damage after freezing and thawing, with a significant decrease in strength, and the strength retention rate was only 70%. Zhang et al. also conducted experimental section studies using a lime content of 5%, and the core samples extracted during core drilling were intact, in good surface condition, and without looseness, with an unconfined compressive strength of 1.78 MPa, meeting the design strength requirements. The rebound modulus of the core samples increased year by year, and the rebound modulus was generally greater than that of ordinary inorganic semi-rigid materials. In the road condition performance test results, the PCI index and IRI value were lower than those of ordinary semi-rigid material pavements, and the flatness distribution was stable.

As shown in Figure 6, compared to previous studies, this research has increased the content of lime and fly ash to improve the strength and actual road performance of the material. The unconfined compressive strength and strength retention rate in durability performance of the material have been significantly enhanced. Among them, the mixed method used in this study has led to a significant increase in the 7-day unconfined compressive strength, resulting in excellent early strength performance. In the test section, the actual road performance has improved significantly compared to the research results of Zhang et al.

### 3.2. Screen Test

**Figure 7:**
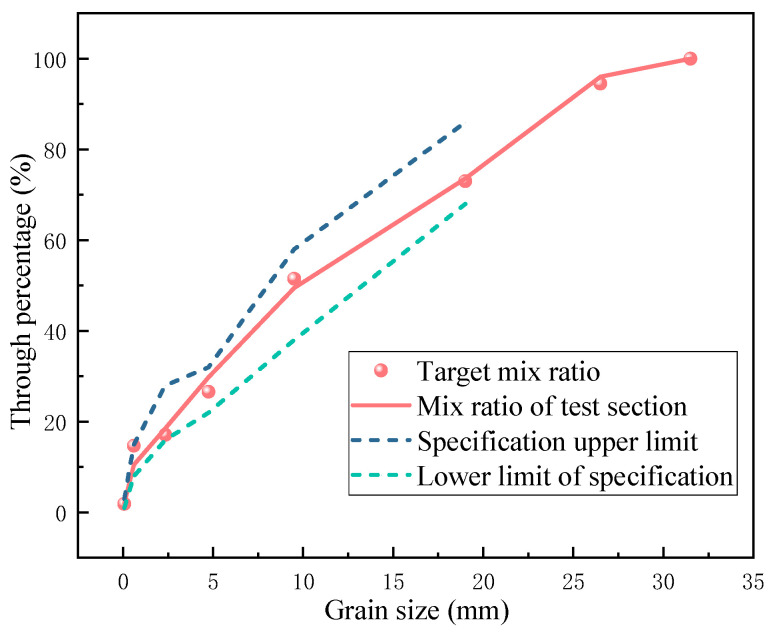
Comparison diagram of grading curve.

The screening results of recycled aggregate used in the test section are shown in Table 1, and the comparison diagram of the grading curve is shown in Figure 7. It can be seen that the particle grading of the construction mix proportion and the target mix proportion in the test section are in good agreement, meeting the specification requirements.

### 3.3. Compaction Test and Ash Dose Test

The test results are shown in Table 2. The average value of the optimal moisture content is 11.2%, and the average value of the maximum dry density is 1.920 g/cm^3^. The test results are shown in Table 3. It can be seen from the table that the ash dosage of the mixture meets the design requirements, and the deviation is within the specified range.

### 3.4. Unconfined Compression Strength Test

**Figure 8:**
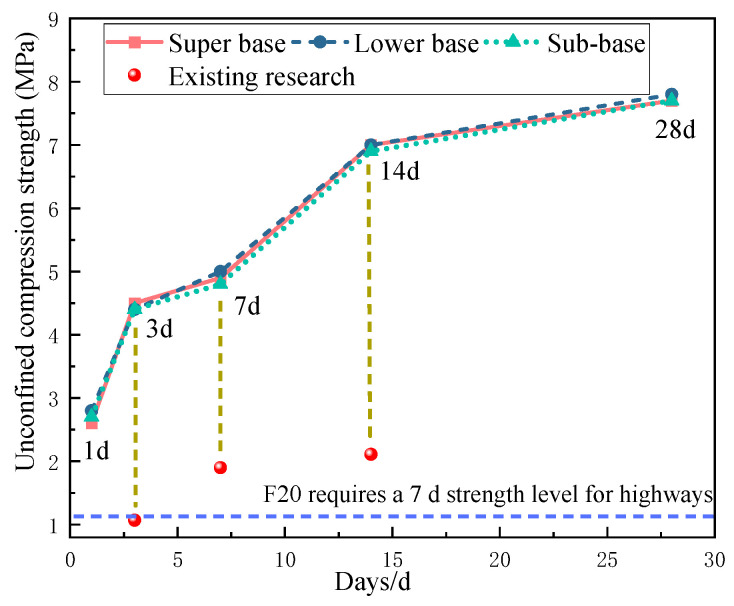
Unconfined compressive strength of the mixture at each age.

The test results are shown in Figure 8. It can be seen from the figure that the material strength development of the three parts of the structure is basically the same. The 7 d unconfined compressive strength of the three-layer structural system far exceeds the technical requirements for highway-grade heavy and extremely heavy traffic specified in the “Technical Specifications for Construction of Highway Pavement Base” (JTG/T F20-2015) [25], demonstrating excellent performance. The material strength of the three parts of the structure is more than 7 MPa at 28 d, and the strength at 28 d is more than 5 MPa higher than that at 1 d. The existing research shown in the figure presents experimental results from previous studies, where the contents of lime and fly ash were 7% and 13%, respectively. Although these meet the specifications, it is evident that the strength results are significantly lower than those of the current test segment.

### 3.5. Frost Resistance Test

**Figure 9:**
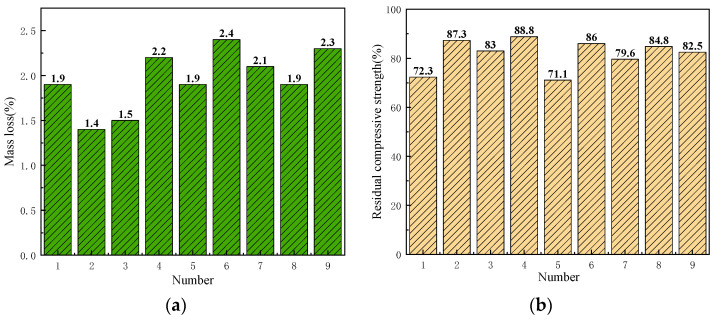
Frost resistance test: (**a**) mass loss; (**b**) residual compressive strength ratio.

Figure 9 shows the mass loss rate and residual compressive strength ratio of the specimen after freeze–thaw. It can be seen from Figure 9a that the mass loss rate of all base course specimens after freezing and thawing is less than 5%, with an average of 2.0%. It can be seen from Figure 9b that the residual compressive strength ratio of the base course specimens after freeze–thaw is greater than 70%, with an average value of 81.7% and good frost resistance.

### 3.6. Pavement Coring Detection

Through the on-site core drilling test, the core samples of the sub-base, lower base, and upper base can be taken out completely twice. Observing the core sample, it is found that the aggregate grading is good, and the core sample is smooth and straight, which basically meets the requirements, indicating that the quality of the lime–fly ash-stabilized recycled aggregate base is good, which can meet the actual use needs. However, some core samples have rotten roots. The main reasons are that the lower part is dry when paving lime–fly ash-stabilized recycled aggregate, without timely watering, and the lower part does not form effective strength. At the same time, large torsion is generated during core drilling, resulting in loose and rotten roots at the lower part of the core sample.

**Figure 10:**
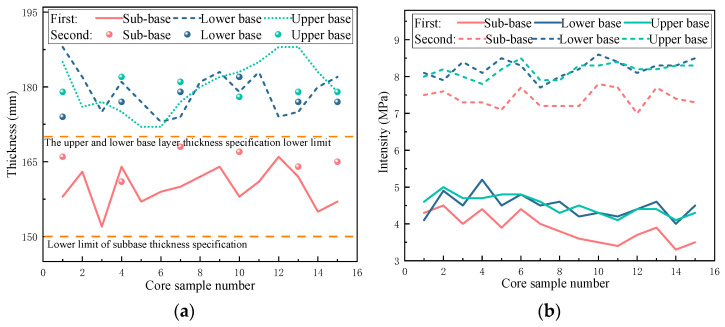
Core detection: (**a**) core thickness detection; (**b**) coring strength test.

The obtained core samples are numbered according to the pile number. The two core samples obtained at the same pile number are the same. After coring, the thickness and strength of the sub-base, lower base, and upper base are measured, respectively. The test results are shown in Figure 10. The average thickness of the sub-base is 162.0 mm, the average thickness of the lower base is 179.0 mm, and the average thickness of the upper base is 179.6 mm. All measured values meet the requirements of the specification and are qualified. The sub-base, lower base, and upper base of the two tests meet the strength design requirements. The average value of the second test of the sub-base is 3.52 MPa higher than that of the first test, the lower base is 3.74 MPa higher, and the upper base is 3.66 MPa higher. The strength of the second coring is kept in the range of 7~9 Mpa, which is consistent with the strength change law of the general lime–fly ash-stabilized base material.

### 3.7. Compaction Detection

**Figure 11:**
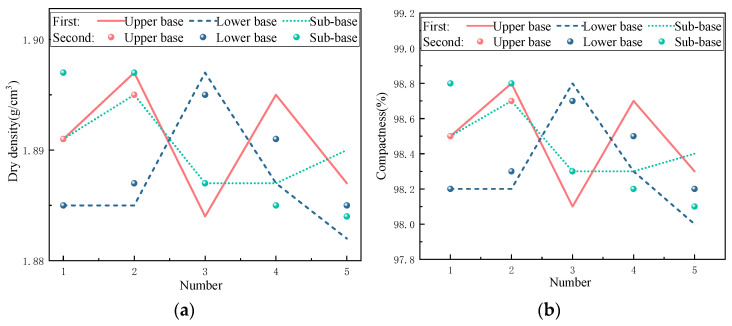
Compaction detection: (**a**) dry density; (**b**) compactness.

As a key indicator of quality inspection, compactness represents the density of the compacted base. The higher the value, the greater the density, and the better the overall performance of the base. The sand-filling method is used to detect the compactness. The previous test data are used for the detection. The maximum dry density is 1.920 g/cm^3^, and the optimal moisture content is 11.2%. The test results are shown in Figure 11. It can be seen from the figure that the compactness of the base course of the test section in the two tests is more than 98%, which meets the test requirements of 95%, indicating that the selected mix proportion and compaction process have a good effect.

### 3.8. Rebound Deflection Detection

Deflection reflects the strength, stiffness, and road state of the pavement structure layer. According to the field test procedure and the test method of t0953-2008 falling weight deflectometer, the results are shown in Table 4. The representative value of roadbed deflection before paving is 219.53 (0.01 mm), which is relatively large. The representative value of the deflection of the lime–fly ash-stabilized recycled aggregate base 9 days after paving is 24.89 (0.01 mm), which is 88.67% lower than that before paving, indicating that the subgrade bearing capacity of the test section is increased and the anti-deformation ability is improved.

## 4. Conclusions

This article studies the road performance of recycled materials from construction waste, relying on the supporting project of the Qingdao Cross-Sea Bridge. This study breaks through the technical bottleneck of low substitution rates of recycled aggregates from construction waste, employing 100% recycled construction waste aggregate instead of natural aggregate to prepare stabilized gravel base materials in the sub-base and base layers of the test section. It enhances the research and discussion on the use of recycled construction waste aggregates in the test section and proposes new mix designs and construction key points for practical applications. First, a series of laboratory tests, including sieving, unconfined compressive strength tests, etc., were conducted to obtain the relevant technical indicators. Then, field tests such as compaction degree and core sampling were conducted according to the specifications, and the technical indicators for the test section were obtained. Finally, a detailed study was conducted on the specific effects of using construction waste, leading to the following conclusions:

1. The particle gradation of the construction mix in the test section matched well with the target gradation. The amount of lime and fly ash meets the design requirements.
2. The development of unconfined compressive strength for the upper layer, lower layer, and base layer was generally consistent. The 7 d unconfined compressive strength of the three-part structural layer far exceeds technical requirements, demonstrating excellent performance.
3. The frost resistance is good, with a mass loss rate of less than 5%, and the residual compressive strength ratio is greater than 70%. Two rounds of core sampling showed that the aggregate gradation was good and smooth, essentially meeting the requirements. Both the thickness and strength meet the design requirements.
4. The compaction degree from both tests on the sub-base layer was over 98%, indicating that the chosen mix design and compaction process were effective.

In conclusion, through the various studies and discussions conducted on the test section, the technical performance and practical road feasibility of the stabilized gravel base materials have been validated, providing a necessary basis for the practical application of using 100% recycled construction waste aggregates to replace natural aggregates in the preparation of stabilized gravel base materials.

## Figures and Tables

**Table 1:** Design grading range.

Mesh Size (mm)	Passing Percentage of Target Mix Proportion (%)	Percentage of Passing Test Section Mix Proportion (%)	Upper Specification Limit (%)	Lower Specification Limit (%)
31.5	100	100	—	—
26.5	94.5	96	—	—
19	73.0	73.6	86.0	68.0
9.5	51.5	49.5	58.0	38.0
4.75	26.6	29.8	32.0	22.0
2.36	17.1	18.6	28.0	16.0
0.6	14.7	10.5	15.0	8.0
0.075	1.9	2.1	3.0	1.0

**Table 2:** Moisture content and dry density of mixture.

Test No	Optimum Moisture Content (%)	Maximum Dry Density (g/cm^3^)
1	11.1	1.919
2	11.2	1.920
3	11.2	1.920
Average value	11.2	1.920

**Table 3:** Test results of mixture ash dosage.

Upper Base (%)	Lower Base (%)	Sub-Base (%)
33.7	33.5	33.4
33.3	33.6	33.6

**Table 4:** Rebound deflection detection results.

Detection Time	Engineering Location	Mean Deflection (0.01 mm)	Standard Deviation (0.01 mm)	Coefficient of Variation (%)	Representative Value of Deflection (0.01 mm)
Before paving	ZK0 + 220-450 roadbed	164.95	42.64	25.85	219.53
9 days after paving	ZK0 + 220-450 base layer	16.19	4.35	26.87	24.89

## Data Availability

The original contributions presented in the study are included in the article, further inquiries can be directed to the corresponding author/s.
